# Bacterial infections and outcomes of inpatients with COVID-19 in the intensive care unit during the delta-dominant phase: the worst wave of pandemic in Iran

**DOI:** 10.3389/fpubh.2024.1411314

**Published:** 2024-09-09

**Authors:** Mojtaba Akbari, Yeganeh Dehghani, Mohammad Shirzadi, Samaneh Pourajam, Majid Hosseinzadeh, Mahdi Sajadi, Malihe Alenaseri, Mansour Siavash, Leila Jafari, Hamid Solgi

**Affiliations:** ^1^Isfahan Endocrine and Metabolism Research Center, Isfahan University of Medical Sciences, Isfahan, Iran; ^2^Amin Hospital, Isfahan University of Medical Sciences, Isfahan, Iran; ^3^Department of Internal Medicine, School of Medicine, Isfahan University of Medical Sciences, Isfahan, Iran; ^4^Department of Genetics and Molecular Biology, School of Medicine Isfahan University of Medical Sciences, Isfahan, Iran

**Keywords:** bacterial infection, multi-drug resistant, COVID-19, intensive care unit, Iran

## Abstract

**Background:**

Epidemiological data regarding the prevalence of bacterial multidrug-resistant (MDR) Gram-negative infections in patients with COVID-19 in Iran are still ambiguous. Thus, in this study we have investigated the epidemiology, risk factors for death, and clinical outcomes of bacterial infections among patients with COVID-19 in the intensive care unit (ICU).

**Method:**

This retrospective cohort study included patients with COVID-19 hospitalized in the ICU of a university hospital in Iran between June 2021 and December 2021. We evaluated the epidemiological, clinical, and microbiological features, outcomes and risk factors associated with death among all COVID-19 patients. Data and outcomes of these patients with or without bacterial infections were compared. Kaplan–Meier plot was used for survival analyses.

**Results:**

In total, 505 COVID-19 patients were included. The mean age of the patients was 52.7 ± 17.6 years and 289 (57.2%) were female. The prevalence of bacterial infections among hospitalized patients was 14.9%, most of them being hospital-acquired superinfections (13.3%). MDR *Klebsiella pneumoniae* and *Staphylococcus aureus* were the most common pathogens causing respiratory infections. Urinary tract infections were most frequently caused by MDR *Escherichia coli* and *K. pneumoniae*. The overall in-hospital mortality rate of COVID-19 patients was 46.9% (237/505), while 78.7% (59/75) of patients with bacterial infections died. Infection was significantly associated with death (OR 6.01, 95% CI = 3.03–11.92, *p*-value <0.0001) and a longer hospital stay (*p* < 0.0001). Multivariate logistic regression analysis showed that Age (OR = 1.04, 95% CI = 1.03–1.06, *p*-value <0.0001), Sex male (OR = 1.70, 95% CI = 1.08–2.70, *p*-value <0.0001), Spo2 (OR = 1.99, 95% CI = 1.18–3.38, *p*-value = 0.010) and Ferritin (OR = 2.33, 95% CI = 1.37–3.97, *p*-value = 0.002) were independent risk factors associated with in-hospital mortality. Furthermore, 95.3% (221/232) of patients who were intubated died.

**Conclusion:**

Our findings demonstrate that bacterial infection due to MDR Gram-negative bacteria associated with COVID-19 has an expressive impact on increasing the case mortality rate, reinforcing the importance of the need for surveillance and strict infection control rules to limit the expansion of almost untreatable microorganisms.

## Introduction

Coronavirus disease 2019 (COVID-19), the disease caused by severe acute respiratory syndrome coronavirus 2 (SARS-Co-V2), was declared a global pandemic in early 2020. The emergence of SARS-CoV-2 has presented a formidable medical challenge to clinicians and health systems (1, 2).

The prevalence of bacterial infections in patients with COVID-19 is associated with morbidity and mortality. The studies revealed that secondary bacterial infections or superinfections are more frequent than co-infections, especially among patients admitted to the intensive care units (ICUs) and those receiving high doses of immunosuppressive drugs (3–5). In Iran and other countries around the world, ICUs have been the main battleground for the treatment of patients with severe COVID-19. Patients with severe COVID-19 admitted to the ICU may require invasive mechanical ventilation, and since they are often exposed to other invasive procedures, they are highly susceptible to secondary bacterial infections. The percentages of bacterial co-infection and superinfection in COVID-19 patients vary widely, ranging from 1 to 8% and 7 to 50%, respectively (6, 7). These patients are more susceptible to developing ventilator-acquired pneumonia (VAP) and bloodstream infections, especially infections caused by multi-drug-resistant (MDR) Gram-negative bacteria (GNB) (7, 8). The aims of this study were (i) to estimate the prevalence of bacterial co-infection and superinfection based on clinical diagnoses and microbiologic testing, and (ii) to describe the clinical and microbiological characteristics of COVID-19 patients with and without bacterial infections in ICU. The impact of co-infections and superinfections on patient outcomes was also assessed.

## Methods

### Study design and setting

This single-center, retrospective cohort study analyzed critically ill patients with COVID-19 pneumonia who were admitted to a 22-bed ICU at an academic hospital in the center of Iran. With the beginning of the fifth wave of the pandemic, which according to the Iran Centers for Disease Control (ICDC) coincided with the spread of the Delta strain, our hospital was exclusively dedicated to the cure of COVID-19 patients. The study was approved by the Isfahan University of Medical Sciences (reference number No. 2401267). Furthermore, it was conducted according to the guidelines of the Declaration of Helsinki.

### Inclusion/exclusion criteria

We included all patients admitted with a positive real-time reverse transcription PCR (RT-PCR) nasopharyngeal or oropharyngeal swab test and/or by fulfilling clinical diagnostic criteria provided during the pandemic peak for SARS-CoV-2 between 13th June 2021 and 14th December 2021. Patients were excluded if they were less than 18 years old, with ICU stay ≤48 h, and duplicated patients and patients who had been transferred to another hospital or were transferred from another hospital. For patients with multiple hospitalizations, only the first was included.

### Data collection

The following information was extracted from the electronic health records: demographic characteristics (age, sex, body mass index), clinical information at hospital admission (signs, symptoms, comorbidities), O2sat at hospital admission, respiratory support (non-invasive and invasive) at ICU admission, laboratory tests at hospital admission [C-reactive protein (CRP), erythrocyte sedimentation rate (ESR), D-dimer and ferritin], type of bacterial infections, susceptibility profile of bacterial pathogens and biological immunosuppressive treatment (tocilizumab, dexamethasone and methylprednisolone).

### Outcomes

This study focused on examining three distinct outcomes. The primary outcome assessed was the occurrence of in-hospital and ICU mortality. Secondary outcomes encompassed the prevalence of bacterial co-infection, secondary bacterial infection, and the requirement for invasive mechanical ventilation.

### Definitions

In the current study, patients with confirmed infection were exclusively included, defined by the presence of a positive bacterial culture of a significant clinical specimen associated with clinical symptoms of infection.

Respiratory infection is defined by a combination of clinical, radiological and laboratory criteria including a significant growth of bacteria in a bronchoalveolar lavage, in a bronchial aspirate or suitable sputum (>25 PMN and < 10 epithelial cells × 100). Ventilator-associated pneumonia (VAP) is a lung infection in a patient mechanically ventilated for >48 h. Urinary infection was defined as the growth of a bacterium in a cultured urine sample from a patient with clinical signs and/or the consideration of such urinary infection as clinically significant by one of our experts. Bloodstream infection (BSI) is defined by the growth of bacteria in at least one blood culture or two positive blood cultures for a common skin contaminant (9).

All of these clinically indicated infections are classified as co-infection or secondary infection. If diagnosis was at the time of or within the first 24 h of hospital admission, these infections were defined as community-acquired infections or co-infections. If diagnosis occurred ≥48 h from admission for COVID-19, these infections were defined as hospital-acquired infections or superinfections. Bacterial isolates were defined as MDR if they were resistant to at least one agent in three or more antimicrobial categories (5).

### Statistical analysis

Statistical analysis was performed using the SPSS software for Windows (version 25, SPSS, Inc., Chicago, IL, United States). Quantitative characteristics are reported as mean ± 1 SD or median [IQR] and Qualitative ones are reported as Number (percent). The Shapiro–Wilk test was used for the normality assessment of quantitative variables. The comparison between patients with and without infection was conducted using various statistical tests such as the independent sample t-test, Mann–Whitney U test, chi-square, and Fisher exact test. Logistic regression analysis was used to conduct both univariate and multivariate analyses, enabling the calculation of crude odds ratios (ORs) and adjusted odds ratios (AORs) for the variables under consideration. The survival rate was estimated using the Kaplan–Meier method and the log-rank test was applied for comparison of survival function. All hypothesis testing was two-tailed and the level of significance was considered to be less than 0.05 in all tests.

## Results

### Description of overall population and infection

**Figure 1:**
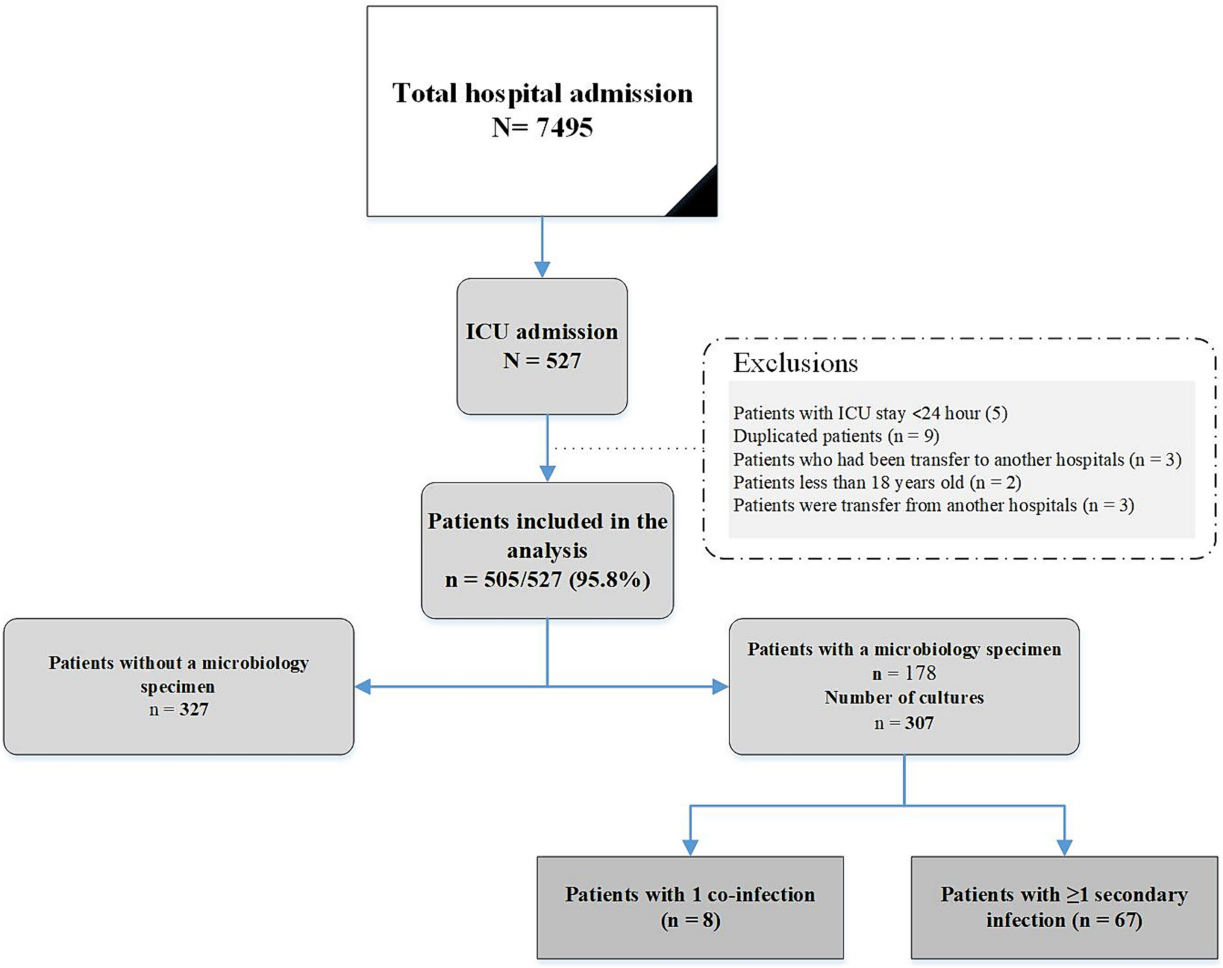
Flow chart of enrolled patients.

During the study period, an evaluation was conducted on 505 consecutive adults with COVID-19 who fulfilled the inclusion criteria. Figure 1 shows the flowchart of patient inclusion. Of these, 289 (57.2%) were female; the mean age was 52.7 ± 17.6 years. Hypertension (120/505, 23.8%) was the most common underlying disease, followed by diabetes mellitus (99/505 19.6%) and chronic heart failure (67/505, 13.3). The most common symptom of COVID-19 infection was dyspnea (435/505 86.1%), followed by cough (316/505 62.6%) and muscular pain (208/505 41.2%). The characteristics of patients with and without infections are shown in Table 1.

**Table 1:** Characteristics of patients hospitalized in ICU with coronavirus disease 19 (COVID-19).

Characteristic	Overall (*n* = 505)	Group		*p*-value
		Infection (*n* = 75)	Without infection (*n* = 430)	
Age (years)	52.7 ± 17.6	54.76 ± 15.93	52.40 ± 17.86	0.283
Sex, Female/Male	289 / 216	48 / 27	241 / 189	0.199
Body mass index, kg/m2	27.6 [25.1–30.5]	28.4 [25.9–31.5]	27.4 [25.0–30.5]	0.106
BMI category				
Normal (18–24.9)	123 (24.4)	16 (21.3)	107 (24.9)	0.711
Overweight (25–29.9)	231 (45.7)	34 (45.3)	197 (45.8)	
Obese (≥30)	151 (29.9)	25 (33.3)	126 (29.3)	
Signs and symptoms at hospital admission				
Dyspnea	435 (86.1)	69 (92.0)	366 (85.1)	0.111
Cough	316 (62.6)	49 (65.3)	267 (62.1)	0.593
Muscular pain	208 (41.2)	31 (41.3)	177 (41.2)	0.978
Anorexia	130 (25.7)	24 (32.0)	106 (24.7)	0.179
Fever ≥38°C	113 (22.4)	10 (13.3)	103 (24.0)	0.042
Headache	29 (5.7)	7 (9.3)	22 (5.1)	0.174
Joint pain	25 (5.0)	4 (5.3)	21 (4.9)	0.777
Nausea or vomiting	29 (5.7)	3 (4.0)	26 (6.0)	0.600
Chest pain	27 (5.3)	5 (6.7)	22 (5.1)	0.578
Sore throat	16 (3.2)	0	16 (3.7)	0.146
Diarrhea	12 (2.4)	1 (1.3)	11 (2.6)	0.447
Chills	59 (11.7)	12 (16.0)	47 (10.9)	0.207
Drowsiness	22 (4.4)	8 (10.7)	14 (3.3)	0.009
Smell and taste disorders	28 (5.5)	0	28 (6.5)	0.014
Nasal congestion and a runny nose	21 (4.2)	2 (2.7)	19 (4.4)	0.754
Comorbidities				
Chronic heart failure	67 (13.3)	11 (14.7)	56 (13.0)	0.699
Chronic kidney disease	27 (5.3)	4 (5.3)	23 (5.3)	0.628
Diabetes mellitus	99 (19.60)	20 (26.7)	79 (14.8)	0.095
Chronic obstructive pulmonary disease	16 (3.2)	3 (4.0)	13 (3.0)	0.718
Malignancies	6 (1.2)	3 (4.0)	3 (0.7)	0.045
Hypertension	120 (23.8)	22 (29.3)	98 (22.8)	0.219
Chronic blood pressure	13 (2.6)	3 (4.0)	10 (2.3)	0.422
Biological immunosuppressive drug				
Tocilizumab	205 (40.6)	48 (60.4)	157 (36.5)	< 0.0001
Dexamethasone	253 (50.1)	41 (54.7)	212 (49.3)	0.391
Methylprednisolone	430 (85.1)	68 (90.7)	362 (84.2)	0.145
Dexamethasone and Methylprednisolone	185 (36.6)	34 (45.3)	151 (35.1)	0.090
Remdesivir	69 (13.7)	5 (6.7)	64 (14.9)	0.056
O2sat at admission	80 [74–86]	77 [70–85]	81 [75–87]	0.005
Low or high-flow nasal cannula	488 (96.6)	72 (96.0)	416 (96.7)	0.728
Bipap	263 (52.3)	51 (68.0)	212 (49.5)	0.003
Mechanical ventilation	232 (45.9)	60 (80.0)	172 (40.1)	< 0.0001
Hospital LOS, day	11.00 [7.00–18.00]	17.00 [9.00–26.00]	10.00 [6.75–17.00]	< 0.0001
Hospital death (all death)	237 (46.9)	59 (78.7)	178 (41.4)	< 0.0001
Of those who died = <7 days	99	14 (23.7)	85 (47.8)	0.001
Of those who died 8–14 days	65	16 (27.1)	49 (27.5)	
Of those who died = > 15 days	73	29 (49.2)	44 (24.7)	
ICU LOS, day	4 [2–9]	10.00 [5.00–15.00]	4.00 [2.00–8.00]	< 0.0001
ICU death (out of all death)	208 (87.8)	53 (89.8)	155 (87.1)	0.654
Of those who died = <7 days	130	21	109	< 0.0001
Of those who died 8–14 days	53	18	35	
Of those who died = > 15 days	25	14	11	
Duration from ward until ICU, day	2.5 ± 3.8	2.7 ± 3.4	2.4 ± 3.8	0.644
ICU Death by (duration time from ward until ICU)	208	53	155	
0 day (the same time) admit day = Enter ICU n_1_ = 156	63 (30.3)	15	48	0.711
1–5 days n_2_ = 287	108 (51.9)	30	78	
6 days and over n_3_ = 62	37 (17.8)	8	29	

Data presented as Mean ± SD, Median [IQR], Number and N (%). *p*-values calculated by Independent sample t-test, Chi-square test, Mann–Whitney U Test, and Fisher exact test. LOS, Length of Stay; ICU, Intensive care unit; O2sat, Oxygen saturation.

As shown in Figure 1, a total of 97 bacterial co-infections and superinfections were diagnosed in 75 of 505 patients. Table 2 details the types and etiologies of the infections identified in patients with COVID-19.

**Table 2:** Detailed microbiological etiology of 97 bacterial co-infection and superinfections occurring in 75 patients with COVID-19 admitted to the intensive care unit.

Type of infection	N (%)	MDR isolates	N (%)
Respiratory tract infections	64 (66)		52/64 (81.2)
*Klebsiella pneumoniae*	39 (60.9)	CR-*Klebsiella pneumoniae*	21 (53.8)
		CR and Col-R-*Klebsiella pneumoniae*	5 (12.8)
*Acinetobacter baumannii*	6 (9.4)	CR-*Acinetobacter baumannii*	6 (100)
*Pseudomonas aeruginosa*	3 (4.7)	–	–
*Escherichia coli*	2 (3.1)	ESBL	2 (100)
*Enterobacter cloacae*	1 (1.6)	CR-*Enterobacter cloacae*	1 (100)
*Staphylococcus aureus*	12 (18.8)	MRSA	5 (41.6)
*Streptococcus pneumoniae*	1 (1.6)	–	–
Urinary tract infections	26 (26.8)		24/26 (92.3)
*Klebsiella pneumoniae*	9 (34.6)	CR-*Klebsiella pneumoniae*	3 (33.3)
		CR and Col-R-*Klebsiella pneumoniae*	3 (33.3)
*Escherichia coli*	11 (42.3)	ESBL	10 (90.9)
*Pseudomonas aeruginosa*	1 (3.8)	–	–
*Enterobacter cloacae*	1 (3.8)	–	–
*Enterococcus faecalis*	4 (15.4)	VRE	1 (25)
Bloodstream infections	4 (4.1)		3/4 (25)
*Klebsiella pneumoniae*	1 (25)	–	–
*Enterobacter cloacae*	1 (25)	CR-*Enterobacter cloacae*	1 (100)
*Staphylococcus aureus*	1 (25)	–	–
*Staphylococcus epidermidis*	1 (25)	–	–
Vaginal	2 (2.06)		2/2 (100)
*Klebsiella pneumoniae*	2 (100)	CR and Col-R-*Klebsiella pneumoniae*	2 (100)
Wound	1 (1.03)		1/1 (100)
*Pseudomonas aeruginosa*	1 (100)	CR-*Pseudomonas aeruginosa*	1 (100)

CR, Carbapenem-resistant; Col-R, Colistisn-resistant, ESBL, Extended-spectrum beta-lactamases; MRSA, Methicillin-resistant *Staphylococcus aureus*; VRE, Vancomycin-resistant Enterococcus.

Respiratory tract infections were the most common (66%), followed by urinary tract infections (26.8%). *Klebsiella pneumoniae* and *Staphylococcus aureus* were the most common causes of respiratory tract infections (60.9 and 18.8%, respectively). Among the UTIs, *Escherichia coli* and *K. pneumoniae* were the most common pathogens (42.3 and 34.6%, respectively).

### Drug resistance profile of isolated pathogens

**Figure 2:**
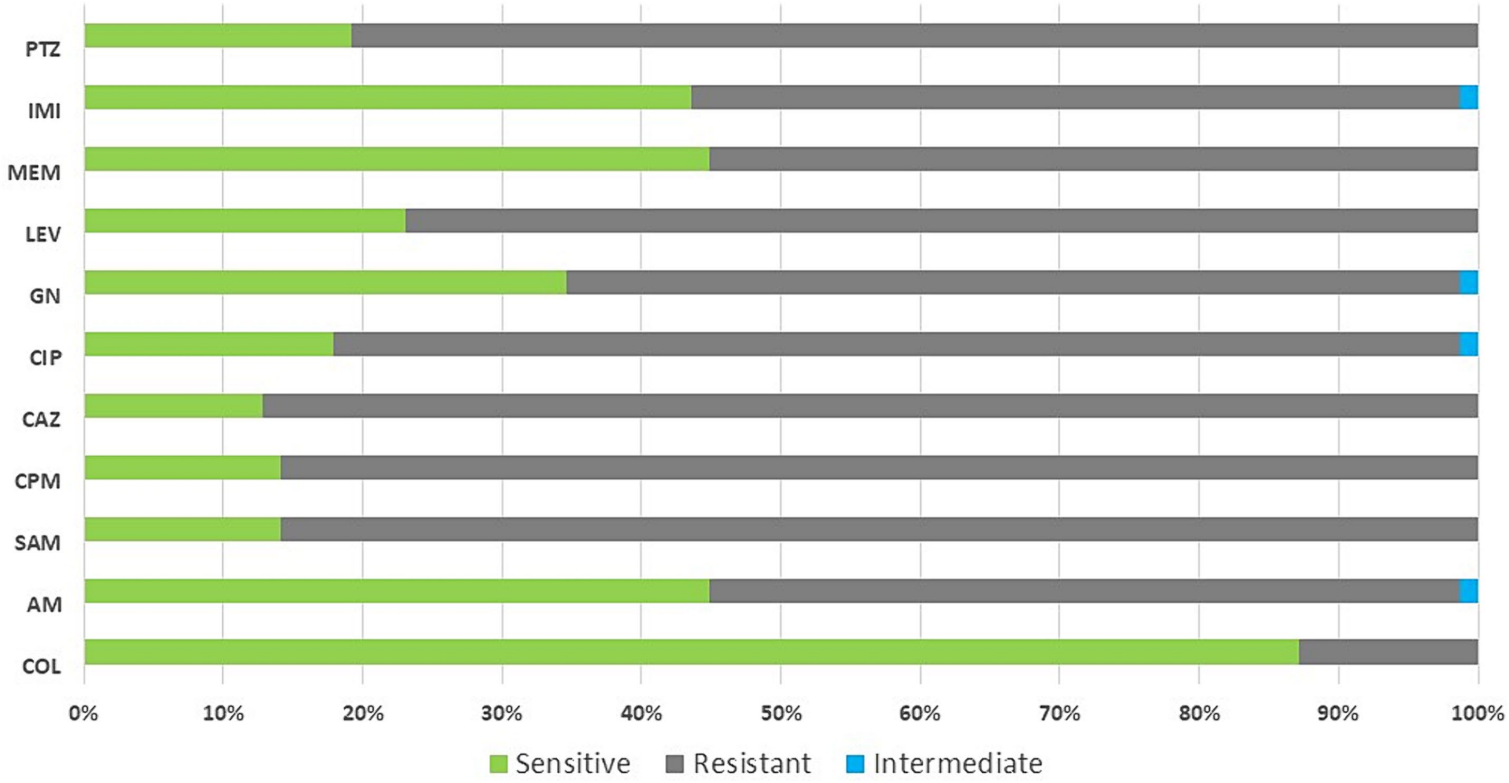
Antibiotic resistance pattern in gram-negative bacteria isolated from patients with COVID-19. Col, colistin; AM, amikacin; SAM, ampicillin-sulbactam; CPM, cefepime; CAZ, ceftazidime; CIP, ciprofloxacin; GN, gentamicin; LEV, levofloxacin; MEM, meropenem; IMI, imipenem; PTZ, piperacillin/tazobactam.

Out of 97 isolates, 84.5% were multidrug-resistance organisms (MDRO). The majority of MDRO belonged to *K. pneumonia*, *E. coli* and *Acinetobacter baumannii*. Overall, 55.1% (43/78) of GNB were resistant to carbapenems (100% (6/6) of *A. baumannii* isolates and 66.6% (34/51) of *K. pneumoniae* isolates). Out of 13 *S. aureus* and four *Enterococcus faecalis* isolates, five and one were methicillin-resistant *S. aureus* (MRSA) and vancomycin-resistant enterococci (VRE) strains, respectively (Table 2). Figure 2 shows the pattern of antibiotic resistance in GNB.

**Overall mortality and associated risk factors**: As shown in Table 1, the overall mortality of the total population was 46.9%. Mortality rate was higher in patients with bacterial infections (78.7% vs. 41.4%, *p*-value <0.0001).

Also, 87.8% of all deaths occurred in the ICU, 89.8% in infection group, and 87.1% in non-infection group; *p*-value <0.0001. As shown in Table 3, bacterial infection was one of the leading causes of mortality. The results revealed that hospital mortality was associated with bacterial infection (Adjusted odds ratio [AOR] = 6.01, 95% CI = 3.03–11.92; *p*-value <0.0001).

**Table 3:** Multivariate logistic regression analysis of in-hospital mortality among patients with COVID-19.

Characteristics	Unadjusted			Adjusted, Model 1			Adjusted, Model 2			Adjusted, Model 3			Adjusted, Model 4		
	OR	95% CI OR	*p*-value	OR	95% CI OR	*p*-value	OR	95% CI OR	*p*-value	OR	95% CI OR	*p*-value	OR	95% CI OR	*p*-value
Age (year)	1.05	1.03–1.06	< 0.0001	1.04	1.03–1.06	< 0.0001	1.04	1.03–1.05	< 0.0001	1.04	1.03–1.05	< 0.0001	1.04	1.03–1.06	< 0.0001
Sex (Male)	2.25	1.57–3.22	< 0.0001	2.04	1.39–2.98	< 0.0001	2.15	1.43–3.23	< 0.0001	2.23	1.48–3.38	< 0.0001	1.70	1.08–2.70	0.023
Obesity status based on BMI															
Normal	1														
Overweight	0.87	0.56–1.35	0.544												
Obese	1.07	0.66–1.72	0.779												
Infection	5.22	2.91–9.37	< 0.0001				6.15	3.26–11.61	< 0.0001	6.22	3.25–11.88	< 0.0001	6.01	3.03–11.92	< 0.0001
Infection type															
UTI	1														
Lung	19.00	4.29–84.13	< 0.0001												
SPO2 (%)	0.94	0.92–0.96	< 0.0001												
SPO2 (≤85%)	3.03	1.99–4.61	< 0.0001				1.98	1.24–3.19	0.005	1.95	1.21–3.14	0.006	1.99	1.18–3.38	0.010
BiPap	2.68	1.87–3.85	< 0.0001												
MV	344.22	154.90–764.96	< 0.0001												
Tocilizumab	1.10	0.77–1.56	0.612							1.25	0.80–1.93	0.317	1.06	0.66–1.68	0.819
Dexamethasone	0.90	0.63–1.28	0.560												
Methylprednisolone	1.74	1.06–2.87	0.029							0.49	0.27–0.89	0.019	0.58	0.30–1.13	0.108
CRP > 40	1.39	0.83–2.32	0.213												
ESR	1.01	1.00–1.01	0.139												
Ferritin	3.61	2.32–5.63	< 0.0001										2.33	1.37–3.97	0.002
D-Dimer	1.63	1.07–2.49	0.023										1.61	0.97–2.67	0.067
RT_PCR	1.33	0.77–2.31	0.307												
Chronic Heart Failure	1.19	0.71–1.99	0.502												
Kidney Heart Failure	1.44	0.66–3.14	0.358												
DM	1.39	0.89–2.16	0.143										0.80	0.45–1.42	0.442
COPD	1.92	0.69–5.38	0.212												
HTN	2.64	1.72–4.04	< 0.0001										1.38	0.78–2.46	0.268
Background Disease (negative)	1														
Background Disease (*n* = 1)	2.54	1.64–3.92	< 0.0001												
Background Disease (*n* = 2)	3.07	1.79–5.28	< 0.0001												
Background Disease (*n* = 3 & over)	1.16	0.48–2.79	0.748												

Model 1, Adjusted for Age and Sex; R^2^ = 0.195. Model 2, Adjusted for all variables in Model 1 as well as Infection and SPO2; R^2^ = 0.299. Model 3, Adjusted for all variables in Model 2 as well as Tocilizumab and Methylprednisolone; R^2^ = 0.311. Model 4, Adjusted for all variables in Model 3 as well as Ferritin, D-Dimer, DM and HTN; R^2^ = 0.353. MV, mechanical ventilation; CRP, C-reactive protein; ESR, erythrocyte sedimentation rate; RT_PCR, reverse transcription polymerase chain reaction; DM, diabetes mellitus; COPD, chronic obstructive pulmonary disease; HTN, hypertension.

**Figure 3:**
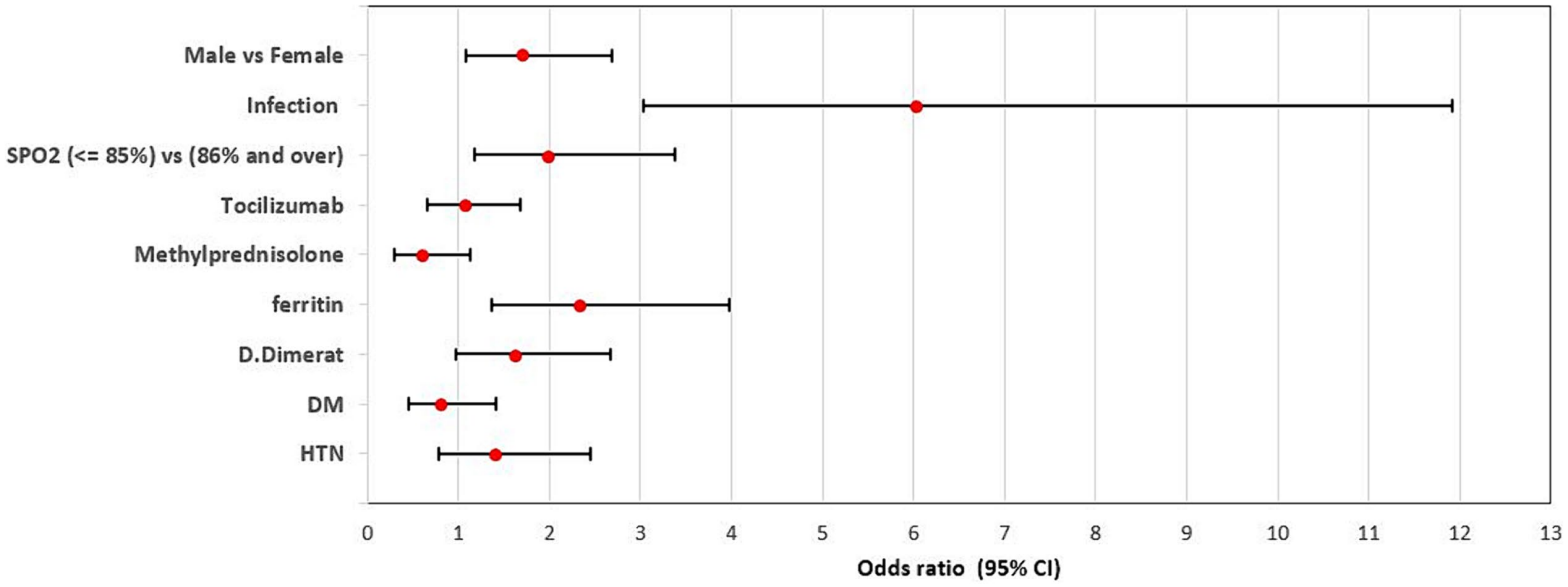
Adjusted odds ratio of factors associated with in-hospital mortality within the group of patients with bacterial infections. Sex male (OR = 1.70, 95% CI = 1.08–2.70, *p*-value <0.0001), Infection (OR = 6.01, 95% CI = 3.03–11.92, *p*-value <0.0001), Spo2 (OR = 1.99, 95% CI = 1.18–3.38, *p*-value = 0.010) and Ferritin (OR = 2.33, 95% CI = 1.37–3.97, *p*-value = 0.002) were independent risk factors associated with in-hospital mortality.

Furthermore, the last model of multivariate logistic regression (model 4) showed that Age (OR = 1.04, 95% CI = 1.03–1.06, *p*-value <0.0001), Sex male (OR = 1.70, 95% CI = 1.08–2.70, *p*-value <0.0001), Infection (OR = 6.01, 95% CI = 3.03–11.92, *p*-value <0.0001), Spo2 (OR = 1.99, 95% CI = 1.18–3.38, *p*-value = 0.010) and Ferritin (OR = 2.33, 95% CI = 1.37–3.97, *p-*value = 0.002) were independent risk factors associated with in-hospital mortality (Table 3; Figure 3).

### Survival analysis

**Figure 4:**
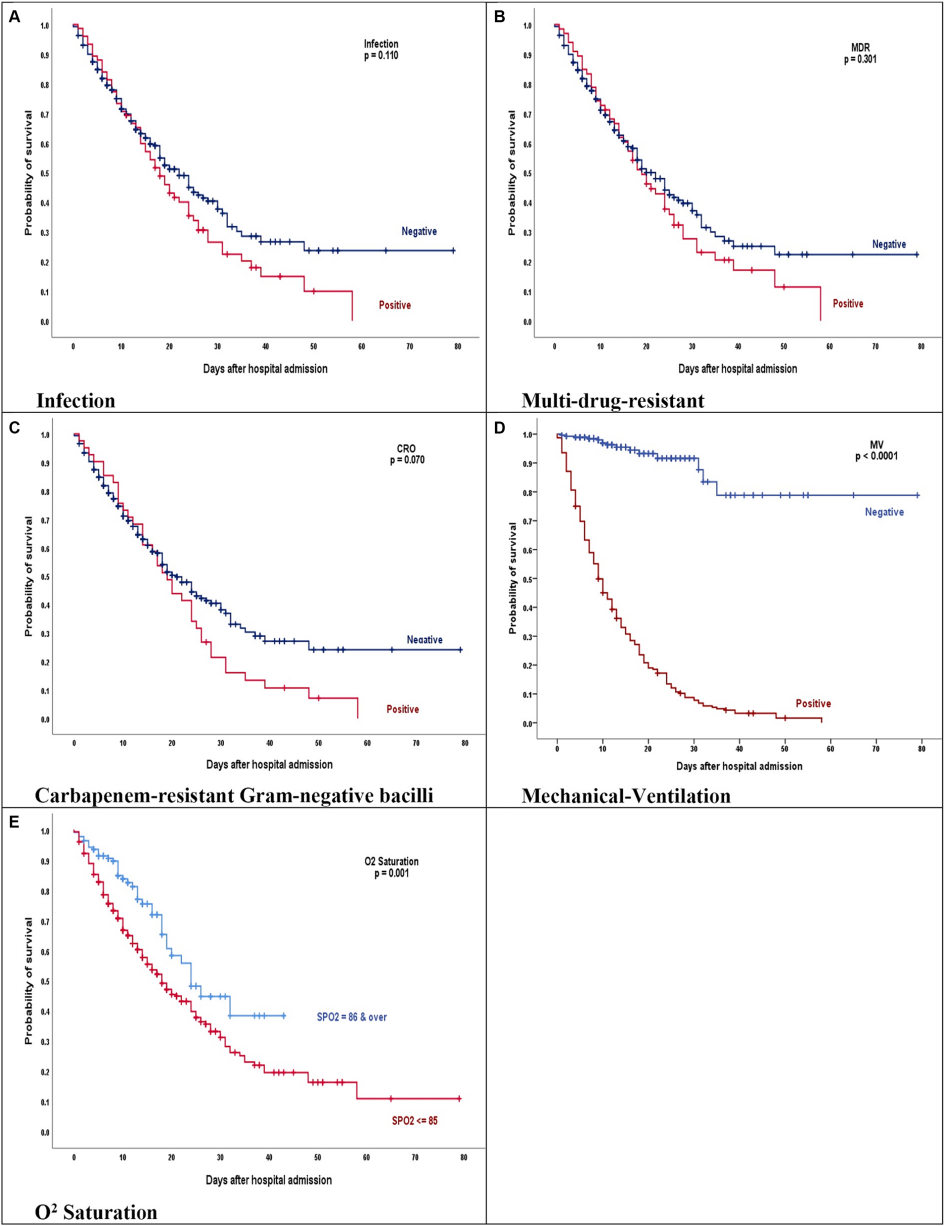
**(A–E)** Kaplan–Meier product limits estimates for probability of survival by **(A)** Infection, *p* = 0.110, **(B)** Multi-drug-resistant, *p* = 0.301, **(C)** Carbapenem-resistant gram-negative bacilli, *p* = 0.070, **(D)** Mechanical-ventilation, *p* = 0.0001, and **(E)** O2 saturation, *p* = 0.001 on mortality. **(A–C)** The red line represents COVID-19 patients who have acquired bacterial infection, MDR and GNB isolates. **(D,E)** The red line represents COVID-19 patients who were intubated and had oxygen less than 85.

Figure 4 shows the survival according to the leading variables. As is shown, Mechanical-Ventilation and O2 Saturation are related to survival.

The Kaplan–Meier curves for these comparisons are presented in Figure 4.

## Discussion

From the beginning of the COVID-19 pandemic in 2019 until the official announcement of the end of the pandemic in July 2023 by the Iranian Ministry of Health and Medical Education, eight waves of the pandemic were reported in Iran. The current study was conducted in the fifth wave of COVID-19, which coincided with the outbreak of the Delta variant.

To our knowledge, this is the first series to specifically characterize the epidemiology, clinical and microbiological presentation, the prevalence of bacterial co-infection and superinfection and the outcome of this cohort of ICU patients in Iran. In this single-center, retrospective cohort study of 505 patients admitted to the hospital with COVID-19, microbiologically confirmed infections were relatively high (75 patients). Co-infection at hospital admission was low and when infections were diagnosed, most were secondary. In this study, we found the prevalence of co-infection was 1.58% (8/505) and 13.3% (67/505) of patients developed a secondary bacterial infection, which was higher than previously published results (8, 10). Yoon et al. considered that the rate of bacterial superinfections of COVID-19 was 30% (32/106), and 12 cases (38%) were associated with multidrug-resistant pathogens (11). The study reported by Bardi and colleagues conducted in Spain found that 40.7% of patients developed a bacterial or fungal nosocomial infection during their ICU stay (6).

It is interesting to note that the highest rate of secondary bacterial infection occurred between the fourth week of July and the first week of August which was associated with MDR pathogens. A study conducted during the first pandemic wave reported a high rate of prevalence of secondary bacterial infection during COVID-19 (7). The isolation of MDR GNB was associated with increased mortality, which is in contrast to previous report (12).

Previous studies revealed that the frequency of co-infection and secondary infection in admitted COVID-19 patients within 48 h of admission was generally low, but the prevalence of secondary bacterial infections has been mainly related to ICU admission, especially with the use of mechanical ventilation; expected epidemiology linked closely to the predominant flora in the hospital (5, 8, 13).

In our study, the rate of MDR GNB infections was relatively high, probably due to the lack of adherence to infection control principles, which causes horizontal transmission among patients. One of the reasons for the spread of these infections among patients was the high number of relocated healthcare personnel in the ICU. Moreover, during this period, infection control programs were strongly directed at avoiding the spread of airborne virus infections, and less attention could have been paid to ordinary infection control practices, and care bundles especially bundle for the prevention of catheter-associated urinary tract infections (CAUTI) and bundle for the prevention of VAP. Unfortunately, we witnessed things such as non-sterile suctions in intubated patients, non-use of gowns and gloves for patients with MDR bacterial infections, and also non-use of isolation rooms in these patients. A very important and interesting thing that was observed from the visits of the microbiologist of the infection control team was that the mouth suction of non-intubated patients with a low level of consciousness was not done cleanly and this work was done with contaminated gloves most of the time which increased the risk of colonization of MDR bacteria in the patient’s mouth, which eventually entered the patient’s lung through aspiration and increased the chance of lung infection. MDR in GNB represents a serious challenge to human health and significantly contributes to global deaths attributable to and associated with bacterial antimicrobial resistance (12). With this regard, secondary bacterial infections caused by MDR bacteria have been increasingly reported during the hospital stays of patients with COVID-19. Infections caused by MDR pathogens are associated with increased mortality and length of hospital stay (5, 12).

Carbapenem-resistant *K. pneumoniae* (CRKP) was the most commonly recovered pathogen from respiratory and urine cultures. It is possible that some of these cases were part of one or more-point outbreaks. Unfortunately, all CRKP isolates were not available for epidemiological typing to enable the investigation of this possibility. The elevated prevalence of CRKP has been highlighted by another study investigating bacterial infection in ICU patients with COVID-19 (7, 12, 14).

Presenting symptoms at hospital admission such as cough, fever, and dyspnea have been widely reported in COVID-19 patients (15, 16). Our findings showed that dyspnea and cough were the most common presenting symptoms in patients.

Regarding the outcome, our results showed that the length of ICU stays and overall mortality rates were significantly higher in COVID-19 patients with bacterial infection than in the non-bacterial group (78.7%) vs. 41.4%. Previous studies have reported that bacterial infections in the ICU were associated with an increase in ICU length of stay and mortality (3, 6, 7).

In the present study, we defined bacterial superinfections based on microbiologic test results, which probably did not represent a true diagnosis of infection because bacterial cultures were not obtained for 327 patients. Nonetheless, we suspect that our findings may have underestimated the true prevalence of bacterial infection.

Previous studies suggested an association between the use of tocilizumab for patients with severe COVID-19 and an increased risk of secondary infections (17, 18). In this report, patients who received tocilizumab had almost twice as many bacterial infections, which is consistent with previous reports.

Furthermore, multivariate logistic regression in patients with both co-infection and superinfection showed that bacterial infection (OR = 5.22; 95% CI, 2.91–9.37; *p*-value <0.0001) nearly 5-fold increased chance of mortality compared to the without bacterial infection group. Similar to our report, studies conducted in Brazil (19), the United States (20), and Italy (4)have shown that bacterial infection increases the risk of death by 5.9, 3.07, and 2.4 times, respectively.

Bacterial lung infections were associated with the highest mortality in our COVID-19 patients, increasing mortality 19-fold (95% CI, 4.29–84.13; *p*-value <0.0001). The prevalence of secondary bacterial lung infection reported in the literature is approximately 15–20%, with higher prevalence reported in patients with COVID-19 admitted to the ICU.

Univariate analysis of laboratory markers revealed that elevated levels of ferritin and D-Dimer were associated with increased mortality, with ORs of 3.61 and 1.63, respectively. High levels of ferritin and D-dimer were reported to be an indicator of poor prognosis and risk of COVID-19 mortality (21).

As reported in previous studies (19, 22), hypertension was associated with a significantly increased risk of in-hospital mortality of COVID-19 patients. Our analysis revealed that hypertension was also associated with increased odds of mortality among COVID-19 patients (OR = 2.64; 95% CI, 1.72–4.04).

Multivariate logistic regression revealed a significant relationship between oxygen saturation below 85% on admission with in-hospital mortality (OR = 3.03, 95% CI = 1.99–4.61, *p*-value <0.0001). Our results indicated that low oxygen saturation on admission was a strong predictor of in-hospital mortality in COVID-19 patients, which is consistent with previous reports (23, 24).

The overall in-hospital mortality rate of patients with COVID-19 was 46.9%, while 95.3% of patients who required IMV died reflecting the difficulty to manage mechanical ventilation of COVID-19 patients during the fifth wave in the context of lack of experience and clear recommendations.

In the present study, the use of ventilators was also a significant risk factor for death. In this study, the hypothesis of anesthesiologists was to use noninvasive respiratory support (NIRS) techniques, including both Biphasic Positive Airway Pressure (BiPAP) and non-invasive Continuous Positive Airway Pressure (CPAP), more often and not intubate patients as much as possible. Our result showed that delaying IMV in COVID-19 patients was associated with increased mortality. Previous studies have reported that in patients not responding to noninvasive respiratory strategies, initial intubation and conversion to IMV were highly recommended (25, 26).

Unadjusted logistic regression analysis showed that the presence of one or two underlying diseases in COVID-19 patients was related to increased mortality.

Our data show that the most widely used biological immunosuppressive therapy was methylprednisolone, which, unlike dexamethasone and tocilizumab, had increased the risk of in-hospital mortality (OR = 1.74; 95% CI, 1.06–2.87; *p*-value = 0.029). Viana et al. (27) reported in their retrospective study that the in-hospital mortality was higher in patients that received prolonged course of corticosteroids (39.5% vs. 26%, *p* < 0.001).

Our study had several limitations. First, it is a single-center retrospective study from a university hospital in the epicenter of the COVID-19 pandemic, which may limit the generalizability of the findings. Second, we were unable to characterize respiratory *Mycoplasma pneumoniae* infections by molecular diagnostic tests (e.g., PCR) and also, urinary antigen tests for pneumococci and *Legionella* sp. were not available. Therefore, we might have missed some bacterial infection. Third, Under-ascertainment of true bacterial infection may also be due to receiving antimicrobials before microbiological sampling, as well as the low sensitivity of culture-based diagnostics in our hospital. Fourth, sending microbial cultures was only done for 35.2% of patients and most of the patients did not have any microbiological sampling, so the prevalence of bacterial infections calculated from our study may not be accurate. Fifth, in this study, antibiotic prescription for patients was reported in general and will be analyzed and discussed in future studies. Finally, due to the low number of bacterial co-infections, we considered both co-infection and superinfection groups as bacterial infections and compared them with the non-infectious group and analyzed them together.

## Conclusion

The role of COVID-19 in favoring bacterial infection is still a matter of debate. Our finding highlights the significant impact of bacterial infection mortality among patients with COVID-19 admitted to ICU. Respiratory tract infections and UTI were the most common bacterial infections due to MDR GNB particularly CRKP emphasizing the need for surveillance and strict infection control strategies to limit the spread of these pathogens, especially in patients with COVID-19 in ICUs.

## Data Availability

The original contributions presented in the study are included in the article/supplementary material, further inquiries can be directed to the corresponding author.
